# Block-Based Connected-Component Labeling Algorithm Using Binary Decision Trees

**DOI:** 10.3390/s150923763

**Published:** 2015-09-18

**Authors:** Wan-Yu Chang, Chung-Cheng Chiu, Jia-Horng Yang

**Affiliations:** Department of Electrical and Electronic Engineering, Chung Cheng Institute of Technology, National Defense University, Taoyuan County 33551, Taiwan; E-Mails: wychang@gmail.com (W.-Y.C.); yang.jiahorng@gmail.com (J.-H.Y.)

**Keywords:** connected components, labeling algorithm, decision tree, scan mask

## Abstract

In this paper, we propose a fast labeling algorithm based on block-based concepts. Because the number of memory access points directly affects the time consumption of the labeling algorithms, the aim of the proposed algorithm is to minimize neighborhood operations. Our algorithm utilizes a block-based view and correlates a raster scan to select the necessary pixels generated by a block-based scan mask. We analyze the advantages of a sequential raster scan for the block-based scan mask, and integrate the block-connected relationships using two different procedures with binary decision trees to reduce unnecessary memory access. This greatly simplifies the pixel locations of the block-based scan mask. Furthermore, our algorithm significantly reduces the number of leaf nodes and depth levels required in the binary decision tree. We analyze the labeling performance of the proposed algorithm alongside that of other labeling algorithms using high-resolution images and foreground images. The experimental results from synthetic and real image datasets demonstrate that the proposed algorithm is faster than other methods.

## 1. Introduction

Connected-component labeling algorithms form the basis of research in areas of computer and machine vision that involve locating objects for visual applications. In real-time applications that analyze the features of detected objects in the background subtraction algorithm, the labeling algorithm classifies the foreground pixels of each group using the connectivity of pixels neighboring the processed pixel. For instance, consider an application that assists object detection over a foreground image. The connected-component labeling algorithm searches for and labels possible candidates by dividing foreground pixels into groups using their eight-connectivity relationship. Once the background subtraction algorithm has segmented all foreground objects from the background of an image, the connected-component labeling algorithm begins its process of locating objects. The labeling algorithm collects and merges pixels into objects by judging the eight-connectivity of the foreground pixels and adjacent neighboring pixels. Following the application of the labeling algorithm, the location, size, and number of foreground objects are ascertained, which helps to determine candidates for object recognition. After finishing the labeling tasks, the objects and their locations are collected and labeled by the connected-component labeling algorithm. Features of the foreground objects can be further extracted by state-of-the-art algorithms such as LBP [1] or HOG [2]. The features can then be used to train a one-class classifier [3,4]. However, when the background subtraction algorithm is applied to high-resolution images, the labeling algorithm has to process more decisions regarding neighboring relationships between foreground pixels in real time. The performance of the labeling process has thus become a new challenge in the field of computer vision.

Several correlations regarding the effect and performance of connected-component algorithms have been proposed in studies on computer vision. The connected-component labeling algorithm was first proposed by Rosenfeld and Pfaltz [5]. Haralick [6] presented a multi-scan labeling algorithm to improve the performance of the labeling process. The multi-scan labeling algorithm resolves neighborhood relationships between the current pixel (*i.e.*, the center pixel of the 3 × 3 pixel block) and neighboring pixels in a scan mask through iterations of forward and backward scans. For each decision made by the scan mask, the provisional labels for the foreground pixels are processed and saved in a temporary array. The performance of the multi-scan labeling algorithm suffers because it involves scanning the entire image more than four times. Lumia *et al.* [7] found that the performance of the methods in [5,6] worsens because of page faults in the virtual memory of the computer. They proposed a combination of an equivalent table and the multi-scan algorithm to reduce memory usage. When scanning to the end of each row in an image, the collected equivalences are solved and updated as provisional labels in another row. The combination algorithm proposed by Lumia *et al.* uses fewer neighbor operations to label foreground pixels, requires four passes over the image, and reduces the execution time by 52.5% compared to [6]. To further improve the multi-scan labeling algorithm, Suzuki *et al.* [8] utilized a lookup table to help resolve the provisional labels, thus reducing the number of scans over an image. This algorithm processes label equivalences using lookup tables during each neighbor operation to eliminate unnecessary memory access. The lookup table efficiently reduces the memory access frequency required to resolve the current pixel and neighboring pixels around the scan mask in multi-scan labeling. Experimental results have shown that the improved multi-scan labeling algorithm reduces the execution time by over 46.5% [7]. To enhance the labeling performance, Wu *et al.* [9] designed binary decision trees for the labeling algorithm. These were intended to eliminate unnecessary neighbor visits by following the decision rules of the scanning sequence in the scan mask. Using binary decision trees, Wu *et al.*’s algorithm requires 23% less time to execute labeling tasks than the method in [8]. He *et al*. [10] and He *et al*. [11] respectively proposed improved labeling methods in the LCS and FCC algorithms, which use two phases of a raster scan with three extra arrays. The three additional arrays, consisting of equivalent label sets and a representative table, are used to resolve label equivalences. Furthermore, the three arrays replace the neighbor operations of each pixel by directly updating the equivalent label sets. The FCC algorithm [11] shortens the labeling time by 93% compared to [8]. He *et al*. [12] proposed the EFS algorithm as a simplified pixel-based scan mask. The EFS algorithm ignores one pixel, saves the state from the last judgment, and generates the Karnaugh map by selecting four pixels, thus deriving two separate procedures. These procedures reduce the decision time by processing only four pixels, thus improving the labeling speed. The EFS algorithm executes the labeling task 9% faster than the method of [11].

He *et al.* [13] designed the RTS algorithm to process the labeling task for pre-recording run data using a two-scan method. The two additional queue data structures are used to record the start and end numbers of foreground pixels over an image before operating labeling tasks. The RTS algorithm uses the recorded queue data to improve the speed of labeling continuous data in the image. When the RTS algorithm is used to label foreground pixels over an image, its execution time is 87% less than that of the approach in [8]. He *et al.* [14] also proposed the ROS algorithm, which is based on preserving the entire run data to improve the labeling tasks. The ROS algorithm requires two additional queues to pre-record all foreground pixels in an image. The ROS algorithm improves the execution time by 72.63% compared to [13] when considering continuous data over an image. However, the ROS algorithm is 2.36% slower than [13] when considering discrete data over an image. He *et al.* [15] developed the ROHS algorithm, which uses one-and-a-half scans for labeling tasks. The ROHS algorithm further utilizes the character of two additional queues to process foreground pixels. The two additional queues index the start and end of all continuous foreground pixels. The ROHS algorithm starts to process the labeling tasks from the start pixel of the current foreground segment after finishing the last foreground segment. When the foreground pixels are pre-recorded by two additional queues, the ROHS algorithm reduces the execution time by 30.58% and 59.02% compared to [11,13], respectively, for a special image type. However, the labeling speed of the ROHS algorithm is 29.41% slower than the method of [11] when the two queues do not pre-fetch the foreground pixels in the special image type. 

As an additional use of the connected-component labeling algorithm, He *et al*. [16] proposed an HCS algorithm to label foreground pixels and closed holes, and compute the Euler number. The HCS algorithm provides the additional hole-counting ability when executing labeling tasks. Two years later, He *et al*. [17] proposed the VF algorithm, which is a pixel-based connected-component labeling algorithm with a 36% faster execution time than HCS [16]. However, when using the VF algorithm to execute labeling tasks, the results and speed are the same as those obtained by the method in [12]. 

The labeling approach proposed by Grana *et al.* [18] uses a larger scan mask of 20 pixels, labeled from *a* to *t*. This 20-pixel block-based scan mask was used to analyze larger areas for each decision in order to minimize memory access. Unlike the pixel-based scan mask, the block-based scan mask utilizes the eight-connected relationship of 3 × 3 pixels to reduce the number of neighbor operations. The block-based scan mask extends the range of consideration, and reduces the required storage for provisional labels, because one label can determine the other three labels in each block. Furthermore, Grana *et al.* [19] proposed the Block-Based Decision Table (BBDT) algorithm, in which OR-decision tables optimize past results relating to binary decision trees by selecting 16 pixels from the 20-pixel scan mask (*i.e.*, excluding pixels *a*, *f*, *l*, and *q*). This block-based scan mask extends the area under consideration for foreground pixels by reducing neighbor operations. Grana *et al.*’s algorithm generates binary decision trees to enhance the labeling speed. The pixels selected for the block-based scan mask and the relevant binary decision trees provide strategies for choosing neighbor operations by analyzing neighboring pixels. The optimized OR-decision table is 51%, 31%, and 35% more efficient at carrying out labeling tasks than the methods of [10,11,18], respectively.

Wu *et al.* [20] proposed the SAUF algorithm for connected-component labeling. SAUF utilizes a binary decision tree to optimize a two-pass labeling algorithm. However, the SAUF algorithm has an execution time that is 39% higher than that of [11]. Using decision tree rules for the block-based algorithm for connected-component labeling [19], Sutheebanjard *et al.* [21] proposed a method that counts the leaf nodes and depth level of the decision tree. Through their analysis and elimination, Sutheebanjard *et al.* proposed an efficient block-based scan mask for the labeling algorithm. Their proposed decision tree contains 86 leaf nodes with 12 depth levels. In a labeling experiment involving an image of 4096 × 4096 pixels, their method required 29.2% and 2.1% less time than the methods proposed in [12,19], respectively. Grana *et al.* [22] adjusted the probabilistic rule weights of the optimal binary decision trees. Through this adjustment, their weighted algorithm achieved an execution time that was 3.4% less than that of the method in [19].

He *et al*. [23] designed the NTS algorithm to reduce the size of the 2 × 2 scan mask from the BBDT algorithm to just 1 × 2 pixels. Thus, the size of a 10-pixel scan mask in the NTS algorithm can ignore three pixels by selecting seven from the scan mask. NTS has a 26% lower execution time than the method in [12]. Furthermore, He *et al*. [24] proposed a CT-based algorithm to improve labeling tasks. Under this approach, next-case decisions are represented as a configuration-transition diagram to obtain better speed. Experimental results for the execution time versus the noise density in images of size 4096 × 4096 pixels showed that the CT-based algorithm reduces the execution time by 26% over that of the method in [19] when the density is equal to 0.5 and the BBDT source code is used in OpenCV [25].

This paper proposes an efficient block-based connected-component labeling algorithm based on three optimization strategies. The scan mask and scan sequence methods in the proposed algorithm differ from those of other labeling algorithms. The first strategy selects eight pixels from the 20 obtained by the scan mask to produce a simplified block-based scan mask. The main purpose here is to reduce redundant memory access to the other 12 pixels. The second strategy switches between two procedures according to the judgment of two pixels on the right side of the current scan in order to reduce the judgment required in the next scan. The third strategy produces two binary decision trees to further optimize the procedure by eliminating redundant neighboring operations in the simplified block-based scan mask. The leaf nodes of the binary decision trees enable the proposed algorithm to determine the neighbor operations for each simplified block-based scan mask. Finally, the second-scan method is performed to update the provisional labels to representative labels.

Two classes of labeling algorithm will not be discussed in this paper. The first class includes the parallel algorithms proposed and studied by Han *et al.* [26]. Such labeling algorithms are not suitable for execution on common workstations. The second class is used for labeling hierarchical images, such as the quadtree implementation described by Samet [27]. The large images are stored in secondary memory and wait to be accessed by the algorithm. As modern computers contain large amounts of main memory, it is not necessary to prohibit the local storage of images. Thus, we exclude these two classes of labeling algorithms from our analysis and comparisons.

The remainder of this paper is organized as follows: in Section 2, we introduce the basic concepts of block-based algorithms involving connected-component labeling. Details of the proposed algorithm, including the simplified block-based scan mask, decision strategies, cases requiring the selection of eight pixels, the two optimization procedures, and the binary decision trees, are described in Section 3. Section 4 presents the results of experiments to test the performance of our proposed algorithm against other connected-component labeling algorithms. We offer our conclusions in Section 5.

## 2. Related Work

**Figure 1:**
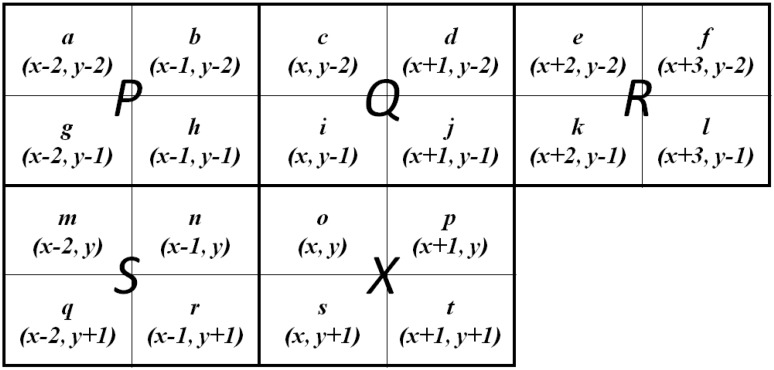
Relative locations of pixels in the block-based scan mask.

In this paper, we focus on the eight-connected relationship in labeling algorithms. Before introducing the proposed algorithm, an introduction to the relevant terminology is given using related work in the area. Let *f*(*x*, *y*) denote the pixel location in an image of resolution *W* × *H*, where 1 ≦ *x* ≦ *W* and 1 ≦ *y* ≦ *H*. The foreground and background pixels of an image are denoted by 1 and 0, respectively. The improved larger scan mask for the pixel-based labeling algorithm with optimized binary decision trees, called the block-based decision table (BBDT), was proposed by Grana *et al.* [12]. The larger scan mask is called the “block-based scan mask,” and includes 20 pixels grouped into four pixels per block. The block is thus a 2 × 2 pixel grid. The BBDT algorithm moves over the 2 × 2 grid using five blocks, as shown in Figure 1. To collect the subsets of foreground pixels with eight-connected neighbor relationships, the BBDT algorithm scans an image twice during the labeling task. In its first-scan method, the BBDT algorithm assigns provisional labels to the foreground pixels, and saves the label equivalences for all subsets in three equivalent arrays. Each judgment of the block-based scan mask considers neighbor operations depending on the state of the neighboring pixels, and follows the decision trees. The number of provisional labels created is reduced by a factor of four. This leads to the generation of subsets in the block-based labeling algorithm. A smaller number of subsets implies fewer neighboring operations and shorter processing time for merge decisions. Having processed label equivalences over an image, the second-scan method is used to update all provisional labels to representative labels using three equivalent arrays.

Pixels *a* to *t* denote the location, text, and meaning from (*x* − 2, *y* − 2) to (*x* + 1, *y* + 1) of each pixel in the image. Blocks *P*, *Q*, *R*, *S*, and *X* denote the location, text, and meaning of the four pixels as a group: (*a*, *b*, *g*, *h*), (*c*, *d*, *i*, *j*), (*e*, *f*, *k*, *l*), (*m*, *n*, *q*, *r*), and (*o*, *p*, *s*, *t*), respectively. In the first-scan method of the BBDT algorithm, 16 pixels are selected (pixels *a*, *f*, *l*, and *q* are excluded, as judgments regarding these can be made using the other pixels in each block). The OR-decision table and decision tree of the 16 pixels are also constructed for each judgment to determine the neighbor operations. The BBDT algorithm uses three equivalence arrays to save the neighbor relationship of each provisional label, much like the two-scan pixel-based labeling algorithm described in [10]. The arrays are used to save and combine label-equivalent sets. The minimal label in the equivalence is called the “representative label,” and is used to update provisional labels when the relevant groups have the common relationship of eight-connectivity. 

In the first-scan method, the raster scan starts from the first pixel and continues until the last pixel, moving from left to right and top to bottom. When applying the raster scan, the BBDT algorithm uses the block-based scan mask to judge the neighbor relationship of the 16 selected pixels. The BBDT algorithm treats the central 2 × 2 pixel-grid as the current block, and discovers neighboring relationships between this block and the other four blocks. The algorithm takes advantage of labeling four pixels at the same time, and changes the scanning range from pixel-by-pixel to block-by-block. Thus, the number of provisional labels is reduced by a factor of four in the first-scan method. The block-based scan mask focuses on the correlation of the block-connected relationship among the five blocks in the scan mask. The foreground pixels are eight-connected, and have the same label if they are in the same block. Considering the block-based scan mask, the BBDT algorithm faces a larger binary decision tree of 20 pixels. The original binary decision trees involve 2^20^ = 1,048,576 cases. By analyzing the 20 pixels in the block-based scan mask, the BBDT algorithm obtains the four necessary central pixels and 12 neighboring pixels for advanced analysis, and bypasses the unnecessary four pixels (*i.e.*, pixels *a*, *f*, *l*, and *q* in Figure 1), thus reducing the number of neighbor operations. Furthermore, the algorithm chooses 192 effective cases to generate OR-decision tables covering 2^16^ = 65,536 cases. The OR-decision table is used with a greedy procedure using a single-entry decision table to produce a binary decision tree containing 211 leaf nodes and 14 depth levels. For a single-entry OR-decision table, the block-based scan mask can directly apply neighbor operations to the relevant foreground pixels in the block-based scan mask. The neighbor operations contain “a new provisional label” (new label), “assign the existing provisional label using the minimal label around the current block” (assign label), “resolve two or three existing provisional labels using the eight-connected relationship” (resolve) (*i.e.*, when two or three blocks are connected to each other, the minimal serial number of provisional labels among those blocks is used to assign and merge their provisional label for consistency), and perform “no operation” [19] to process the neighbor relationships among the five blocks. The OR-decision tables of the BBDT algorithm for the 16 pixels accelerate the decision making process regarding neighbor operations in each situation. The algorithm provides provisional labels for the foreground pixels by following the binary decision trees. The three equivalence arrays are simultaneously refreshed according to the correspondence of the provisional labels in the image.

Following the first-scan method of the BBDT algorithm, the second-scan method begins updating the provisional labels to the representative labels for all foreground pixels in the image. The method updates the provisional label block by block, and sequentially checks the four pixels of the current block against the first pixel in each block.

## 3. Block-Based Connected-Component Labeling Algorithm Using Binary Decision Trees

Our proposed algorithm uses the characteristics of block-connected relationships and the sequential raster scan to simplify the checking of pixels in the block-based scan mask. Unlike the BBDT algorithm, which checks this procedure and the decision tree using 16 pixels for each judgment, the proposed algorithm designs two procedures and binary decision trees in different block-connected situations. The proposed algorithm observes and chooses the necessary pixels from the 20 pixels obtained in the block-based scan mask according to block-connected relationships. It examines the block connectivity among blocks *P*, *Q*, *R*, *S*, and *X* to select the pixels necessary for labeling tasks. For the simplified block-based scan mask, the proposed algorithm determines the block-based relationship between the current block *X* and the other blocks *P*, *Q*, *R*, and *S* to minimize the use of the block-based scan mask. In checking pixels in blocks *P* and *X* of the block-based scan mask, the algorithm considers the connectivity of pixels *o* and *h*. The other pixels in block *P* are bypassed, because the connectivity of blocks *P* and *Q*, or of *P* and *S*, has dealt with neighboring processes in the previous block-based scan mask process. The subsequent selection of the necessary pixels is judged on the neighbor relationship between blocks *Q* and *X*, which requires pixels *i*, *j*, *o*, and *p*. Pixels *c* and *d* should be excluded from the simplified block-based scan mask. In selecting the necessary pixels, the proposed algorithm considers the neighbor relationship between blocks *R* and *X*. Pixels *k* and *p* are selected to join the simplified block-based scan mask. Pixels *f* and *l* cause unnecessary memory accesses in each judgment. The next selection focuses on pixel *e*, which is also bypassed by the simplified block-based scan mask because it has no connection with block *X*. The connectivity of blocks *S* and *X* is judged using pixels *n*, *r*, *o*, and *s*. Pixels *m* and *q* are bypassed because the neighbor operations concerning these have been processed in the previous block-based scan mask. Thus, our algorithm selects the 10 pixels *h*, *i*, *j*, *k*, *n*, *o*, *p*, *r*, *s*, and *t* from the 20 obtained in the block-based scan mask.

**Figure 2:**
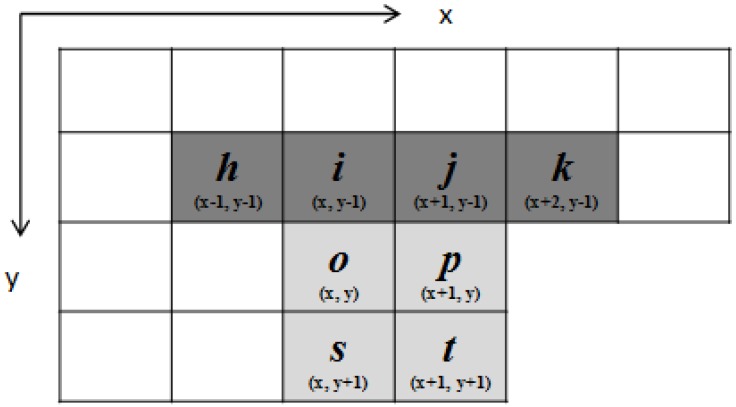
Block-based scan mask of the proposed algorithm.

To simplify further, our proposed algorithm uses two procedures for different block-connected relationships. We design two checking sequences and two resulting binary decision trees, called “procedure 1,” and use “procedure 2” to apply suitable block-connected relationships individually. In terms of the block-connected relationship, the connectivity between blocks *S* and *X* is judged according to pixels *n*, *r*, *o*, and *s*. When pixels *n* and *r* are background pixels, the proposed algorithm directly limits the consideration of the block-connected relationship between block *X* and blocks *P*, *Q*, and *R* in procedure 1. Otherwise, the proposed algorithm considers block-connected relationships between block *X* and blocks *S*, *P*, *Q*, and *R* in procedure 2. Of the 10 checking pixels, pixels *n* and *r* are only used to choose the procedure for the current judgment. Procedure 1 or procedure 2 is selected for the current judgment according to the state of pixel *n* or *r*. When pixel *n* or *r* exists in the current judgment, procedure 2 is utilized to judge the block-connected relationships of block *X* with blocks *S*, *P*, *Q*, and *R*. Otherwise, the judgment uses procedure 1 to reduce the number of relationships that need be considered, thus enhancing performance. Furthermore, the existence of pixels *n* and *r* allows the results for pixels *p* and *t* from the previous judgment to be used. Thus, the judgment of pixels *n* and *r* is simplified by the previous judgment. To summarize, the proposed algorithm judges the block-connected relationships using the eight selected pixels *h*, *i*, *j*, *k*, *o*, *p*, *s*, and *t* by applying two different procedures. The block-based scan mask of the proposed algorithm is shown in Figure 2.

### 3.1. Procedure 1 of the Proposed Algorithm

**Figure 3:**
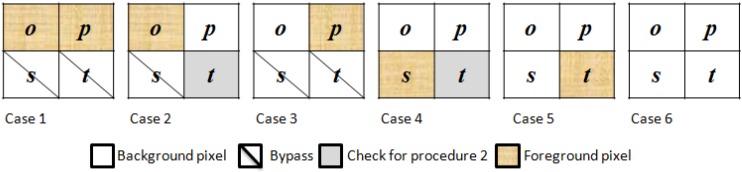
Six selected cases of the current block for procedure 1.

When pixels *n* and *r* are both background pixels, the proposed algorithm chooses procedure 1 to determine the action for the current block *X*. Procedure 1 is designed to judge the block-connected relationships between block *X* and blocks *P*, *Q*, and *R*. The state of pixels *n* and *r* indicates the existence of block *S*. Thus, block *S* has no connection with block *X*. Block *X* then considers fewer block-connected relationships, which enhances the performance of labeling tasks. For procedure 1, the proposed algorithm sequentially checks the pixels of block *X* against pixels *o* to *t*. According to the values of the four pixels in the current block, there are 16 cases for pixels *o*, *p*, *s*, and *t*, which are denoted in binary digits as (*opst*)_2_. (*opst*)_2_ ranges from (0000)_2_, (0001)_2_, (0010)_2_ … to (1111)_2_. The proposed algorithm uses six cases, and simplifies the 10 redundant cases using the binary decision trees of procedure 1. The six cases can determine the final action for the current block depending on the binary number (*hijk*)_2_ of pixels *h*, *i*, *j*, and *k* by following the binary decision tree. (*hijk*)_2_ ranges from (0000)_2_, (0001)_2_, (0010)_2_ … to (1111)_2_. The six cases of procedure 1 in the proposed algorithm are summarized in Figure 3.

**Figure 4:**
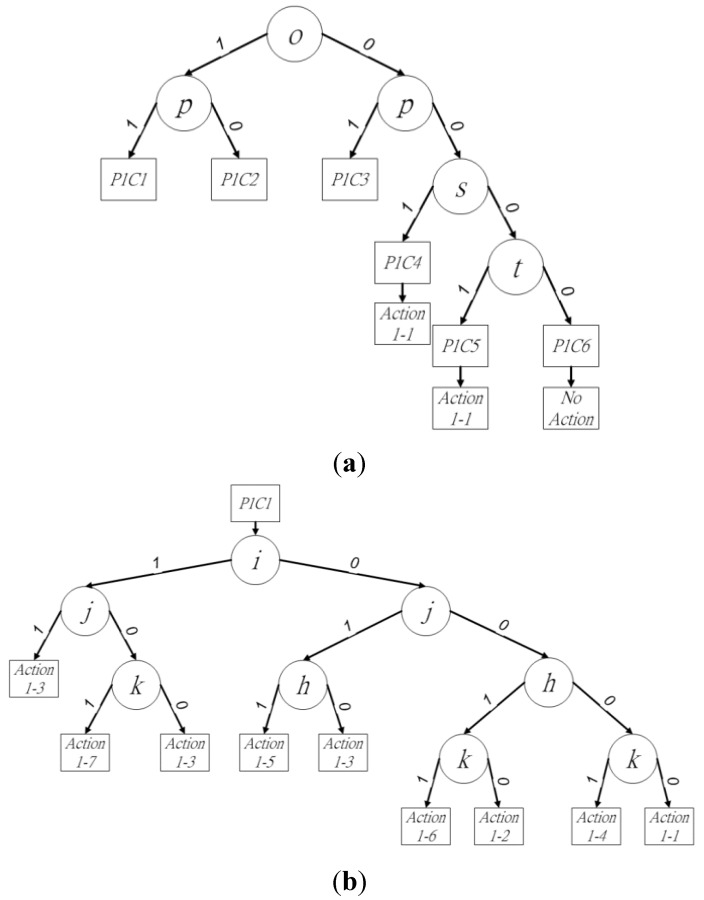

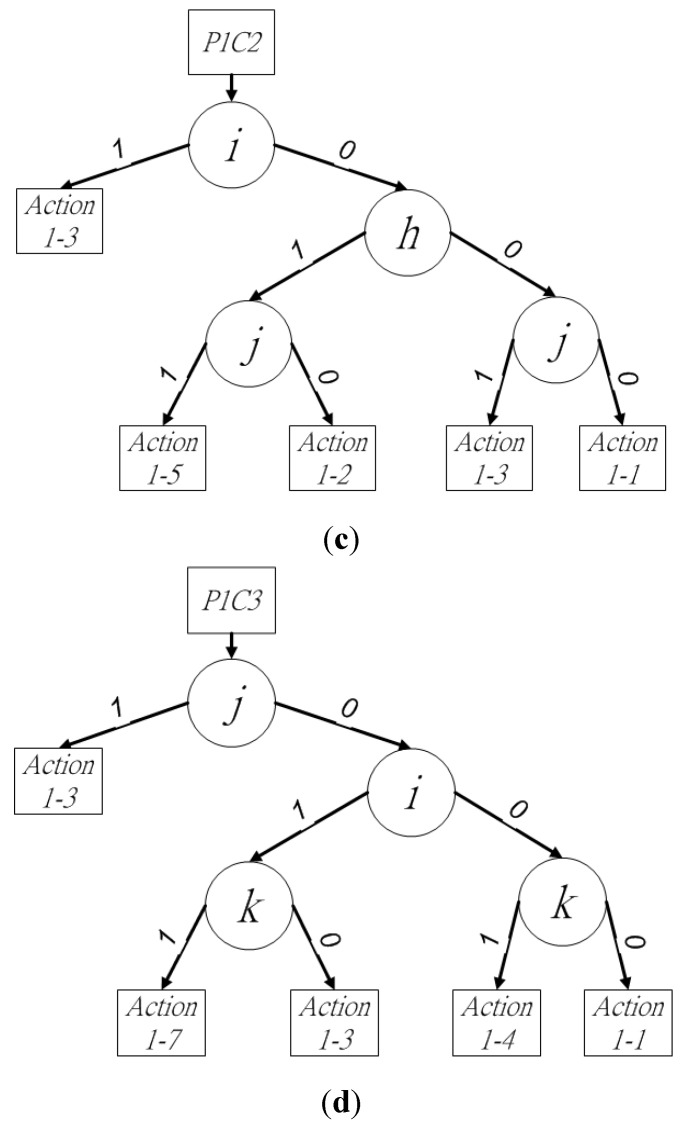
Binary decision tree of (**a**) procedure 1, (**b**) *P1C1*, (**c**) *P1C2* and (**d**) *P1C3.*

The binary decision trees of procedure 1 are shown in Figure 4a. In the six cases for procedure 1, pixels *o* and *p* determine three cases and affect the other three, because the existence of pixels *o* and *p* determines the connectivity between block *X* and blocks *P*, *Q*, and *R*. Thus, these two pixels should be checked first to reduce redundant checking in the current block. To define the binary decision trees, case *w* in procedure *u* is denoted as *PuCw* in this paper, where 1 ≦ *u* ≦ 2 and 1 ≦ *w* ≦ 7.

In Figure 4a, the state of pixel *o* is checked for the existence of the next leaf node. Following this, pixel *p* is checked to choose the next leaf node. When the state of (*op*)_2_ is (11)_2_, (10)_2_, or (01)_2_, the leaf node of *P1C1*, *P1C2*, or *P1C3* is used to judge the neighbor relationships between block *X* and blocks *P*, *Q*, and *R*, as shown in Figure 4b–d, respectively. The state of pixel *s* as either foreground or background determines whether leaf node *P1C4* is used or another leaf node should be checked. Pixel *t* is checked last in the current block *X* for the final leaf node *P1C5* or *P1C6*, when pixels *o*, *p*, and *s* are in the state (000)_2_. For procedure 1, the six cases trigger seven different actions as leaf nodes in the binary decision trees. When the final leaf node of the binary decision tree has been obtained, the proposed algorithm follows the assigned actions for the neighbor operations. This step summarizes and assigns numbers to the final actions, including “a new provisional label,” “assign provisional label to block *P*, *Q*, or *R*,” and “assign provisional label to block *P* or *Q*, and then merge blocks *P* and *Q*, *P* and *R*, or *Q* and *R*” in procedure 1. Action *w* in procedure *u* is denoted as *u-w* in this paper, where 1 ≦ *u* ≦ 2 and 1 ≦ *a* ≦ 11. The necessary neighbor operations of each action in procedure 1 are summarized in Table 1.

**Table 1:** Actions of procedure 1.

Action Number	Neighbor Operations
1-1	Plus serial number and New a provisional label for block *X* as temporary label
1-2	Assign the same provision label as block *P* for the current block *X*
1-3	Assign the same provision label as block *Q* for the current block *X*
1-4	Assign the same provision label as block *R* for the current block *X*
1-5	Assign the same provision label as block *P* for the current block *X.* Resolve the confliction with block *Q* by assigning the provisional label of block *P*
1-6	Assign the same provision label as block *P* for the current block *X.* Resolve the confliction with block *R* by assigning the provisional label of block *P*
1-7	Assign the same provision label as block *Q* for the current block *X.* Resolve the confliction with block *R* by assigning the provisional label of block *Q*

In *P1C1*, the state of pixels *o* and *p* is (11)_2_. The block-connected relationships between block *X* and blocks *P*, *Q*, and *R* should be checked, which requires pixels *h*, *i*, *j*, and *k* to be considered*.* The provisional label for the current block can be determined by following the binary decision sub-tree in Figure 4b. Depending on the locations of all foreground pixels, action numbers 1-1 to 1-7 can be performed by the final leaf node. For the leaf nodes of the binary decision sub-tree, the proposed algorithm employs the strategy of “divide and conquer,” which ensures the connectivity of block *X* with blocks *P*, *Q*, and *R* for the elimination of redundant checks in the proposed algorithm. Block *Q* should first check among blocks *P*, *Q*, and *R* to reduce redundant judgments. Pixels *i* and *j* are checked first, because they indicate the existence of block *Q* and its connectivity with block *X*. Furthermore, pixels *h* and *k* indicate the connectivity of blocks *P* and *R*, respectively, with block *X*. Thus, pixels *i* and *j* are first checked to ensure the existence of block *Q*. The states of pixels *i* and *j* can be (11)_2_, (10)_2_, (01)_2_, or (00)_2_. The four states produce different actions for the current block *X*. When pixels *i* and *j* are (11)_2_, the relationships between blocks *X*, *P*, *Q*, and *R* can be described simply by assigning the provisional label for block *X* using block *Q* as action 1-3. The state (11)_2_ of pixels *i* and *j* implies the existence of block *Q*. Block *P* or *R* must be connected with block *Q* in the previous two rows when either pixel *h* or *k* is the foreground pixel, respectively. Blocks *P*, *Q*, and *R* retain the same provisional labels. Thus, the current block *X* directly assigns the provisional label to block *Q*. When pixels *i* and *j* are in state (10)_2_, the leaf node of the decision tree performs either action 1-7 or 1-3, because blocks *Q* and *R* may have different provisional labels. When pixel *k* is the foreground pixel, the neighbor operations assign the provisional label of block *Q* to *X*, and then merge blocks *Q* and *R* because they are connected by block *X*. Otherwise, action 1-3 assigns the provisional label of block *Q* to *X.* The pixels *i* and *j* are in the state (01)_2_, which triggers either action 1-5 or 1-3, because the disconnection between pixels *h* and *j* means that blocks *P* and *R* may not have the same provisional labels. In this state, blocks *P* and *Q* should be merged by action 1-5 when pixel *h* is the foreground pixel. The neighbor operation following action 1-5 assigns the provisional label of block *Q* to *X*. Otherwise, action 1-3 assigns the provisional label for block *Q* to the current block *X*. The final state of pixels *i* and *j* is (00)_2_. This state triggers four different actions, 1-1, 1-2, 1-4, and 1-6, depending on the four states of (*hk*)_2._ Pixels *h* and *k* represent blocks *P* and *R*. The four states of (*hk*)_2_ indicate the existence of blocks *P* and *R*. The first state of (*hk*)_2_ is (11)_2_. The neighbor operations assign the provisional label of block *Q* to block *X*, and then merge blocks *P* and *R* as action 1-6*.* The second state of (*hk*)_2_ is (10)_2_, in which only block *P* exists in the upper row of the current block *X*. The neighbor operation assigns the provisional label of block *P* to *X* as action 1-2. The next state of (*hk*)_2_ is (01)_2_. In the upper row of block *X*, only block *R* exists*.* The provisional label of block *R* is assigned to the current block *X* as action 1-4. The final state of (*hk*)_2_ is (00)_2_. The neighbor operation requires a new provisional label for block *X*, as action 1-1. Because the provisional label has already been assigned by the leaf node of *P1C1*, pixels *s* and *t* are bypassed to improve labeling performance. The bypassing of pixels *s* and *t* simplifies the consideration of the four cases into one in the current block of procedure 1. At the end of *P1C1*, the binary decision tree of the next judgment chooses procedure 2, because pixel *p* is the foreground pixel representing the existence of block *Q*. 

*P1C2* occurs when pixels *o* and *p* are in state (10)_2_. The block-connected relationships between block *X* and blocks *P* and *Q* should be checked, which requires pixels *h*, *i*, and *j* to be examined*.* The provisional label for the current block can be determined by following the binary decision sub-tree in Figure 4c. The first pixel to be checked is the middle pixel among *h*, *i*, and *j*. Thus, when pixel *i* is the foreground pixel, action 1-3 is performed, because pixels *h* and *j* have the same provisional label as *i*. Otherwise, pixels *h* and *j* are checked. They have four different states. The first state is (11)_2_ for (*hj*)_2_. This indicates the existence of blocks *P* and *Q*, but they may have different provisional labels because of the disconnection between pixels *h* and *j*. Action 1-5 is used as the neighbor operation. The second state of (*hj*)_2_ is (10)_2_, in which only block *P* exists above block *X*. Action 1-2 is thus applied to assign the provisional label of *P* to *X*. The third state of (*hj*)_2_ is (01)_2_, representing the existence of block *R* in the upper row of block *X*. The provisional label of block *X* is assigned as the label of block *R* by action 1-3. The final state of (*hj*)_2_ is (00)_2_, whereby a new provisional label is required by block *X* as action 1-1. At the end of *P1C2*, it is necessary to check pixel *t* to determine between procedures 1 and 2 for the next judgment. Pixel *s* should be bypassed, because the provisional label of the current block *X* has been determined by the aforementioned states. 

In *P1C3*, pixels *o* and *p* are in state (01)_2_. The block-connected relationships between block *X* and blocks *Q* and *R* should be checked, which requires the consideration of pixels *i*, *j*, and *k.* The provisional label for the current block *X* can be determined by following the binary decision sub-tree in Figure 4d. As in *P1C2*, the middle pixel of *i*, *j*, and *k* is first checked. Thus, action 1-3 is directly applied when pixel *j* is the foreground pixel, because pixels *i* and *k* have the same provisional label as *j*. Otherwise, pixels *i* and *k* are checked. Pixels *i* and *k* have four different states. The first state is (11)_2_ for (*ik*)_2_. Blocks *Q* and *R* may have different provisional labels because of the disconnection between pixels *i* and *k* in the first state. Action 1-7 is thus applied for the neighbor operations of assigning the provisional label of block *Q* to *X*, and merging blocks *Q* and *R*. The second state of (*hj*)_2_ is (10)_2_. Action 1-3 is directly applicable here, because only block *Q* exists above the current block *X*. The next state of (*hj*)_2_ is (01)_2_. Action 1-4 is performed for the current block *X*, because blocks *P* and *Q* do not exist above *X*. At the end of *P1C3*, pixels *s* and *t* are bypassed, because the provisional label of block *X* has already been assigned by the binary decision tree. Because block *S* exists in the following judgment, procedure 2 is chosen next.

In *P1C4*–*P1C6*, block *X* has no connection with blocks *P*, *Q*, and *R*. In *P1C4*, pixels *s* and *t* are (11)_2_ or (10)_2_. Action 1-1 is used to obtain a new provisional label for block *X*. When pixel *p* is a background pixel, pixel *t* should be checked to ensure the existence of block *S* for the next judgment and choose the appropriate procedure. *P1C5* of the current block only has one foreground pixel at location *t*. Action 1-1 is therefore utilized to obtain a new provisional label for block *X*. Pixel *t* determines that procedure 2 is chosen in the next judgment. The final case is *P1C6*, in which there are no foreground pixels in block *X*. In this case, all neighbor operations should be bypassed, and the binary decision tree of procedure 1 should be chosen for the next judgment. The pseudo-code for procedure 1 of the first scan in the proposed method is given in Algorithm 1.

**Algorithm 1** Procedure 1 of the First-Scan Method in the Proposed Algorithm

**Begin**
1.    if(o!=0){
2.            if(p!=0)
3.            {P1C1;}
4.            else
5.            {P1C2;}
6.    }else if(p!=0)
7.            {P1C3;}
8.    else if(s!=0)
9.            {P1C4;}
10.   else if(t!=0)
11.           {P1C5;}
12.   else
13.           {P1C6;}
**end**


### 3.2. Procedure 2 of the Proposed Algorithm

**Figure 5:**
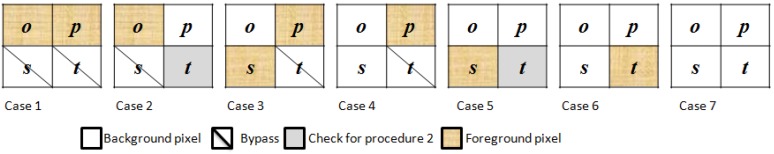
The seven selected cases of the current block for procedure 2.

When pixel *n* or *r* is the foreground pixel in the current judgment, the proposed algorithm chooses procedure 2 to determine the actions for block *X*. There are seven different cases in procedure 2, *P2C1*–*P2C7*, as shown in Figure 5. All seven cases must consider the block-connected relationship with block *S*. The judgments and actions of procedure 2 become more complex than those in procedure 1. Considering *P2C1* as an example, the neighbor operations are “assign the same provisional label as block *S* to block *X*, and then resolve the conflict among blocks *S*, *Q*, and *R*” when the state of (*ijk*)_2_ is (101)_2_. To compare the same locations of the foreground pixel in *P1C1*, *P2C1* must deal with conflicts regarding the provisional labels of blocks *S*, *Q*, and *R*. Although the leaf nodes and the depth levels of the binary decision tree in *P2C1* are the same as those of *P1C1*, the final actions of *P2C1* are more complex.

**Figure 6:**
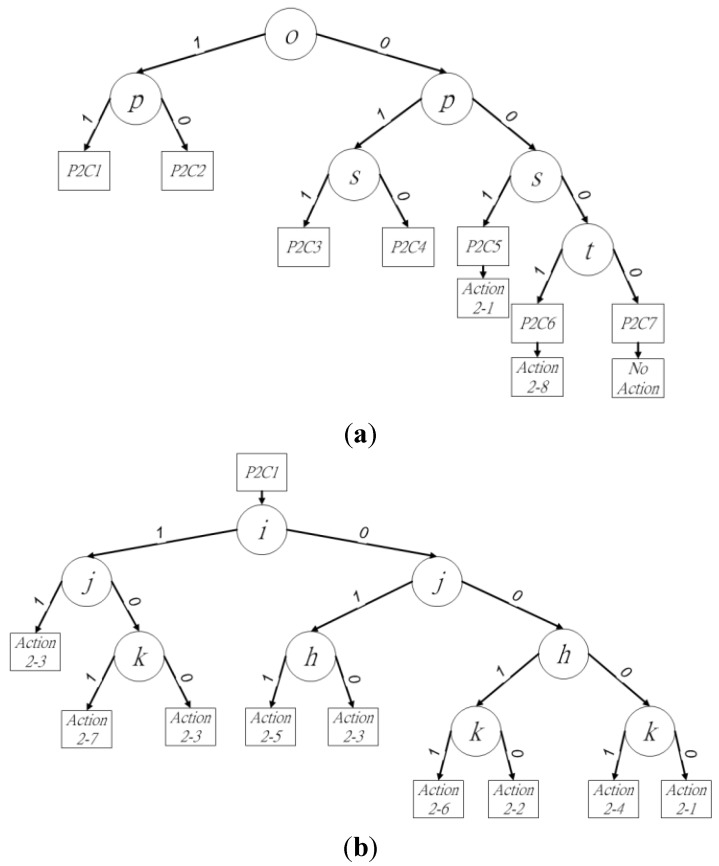

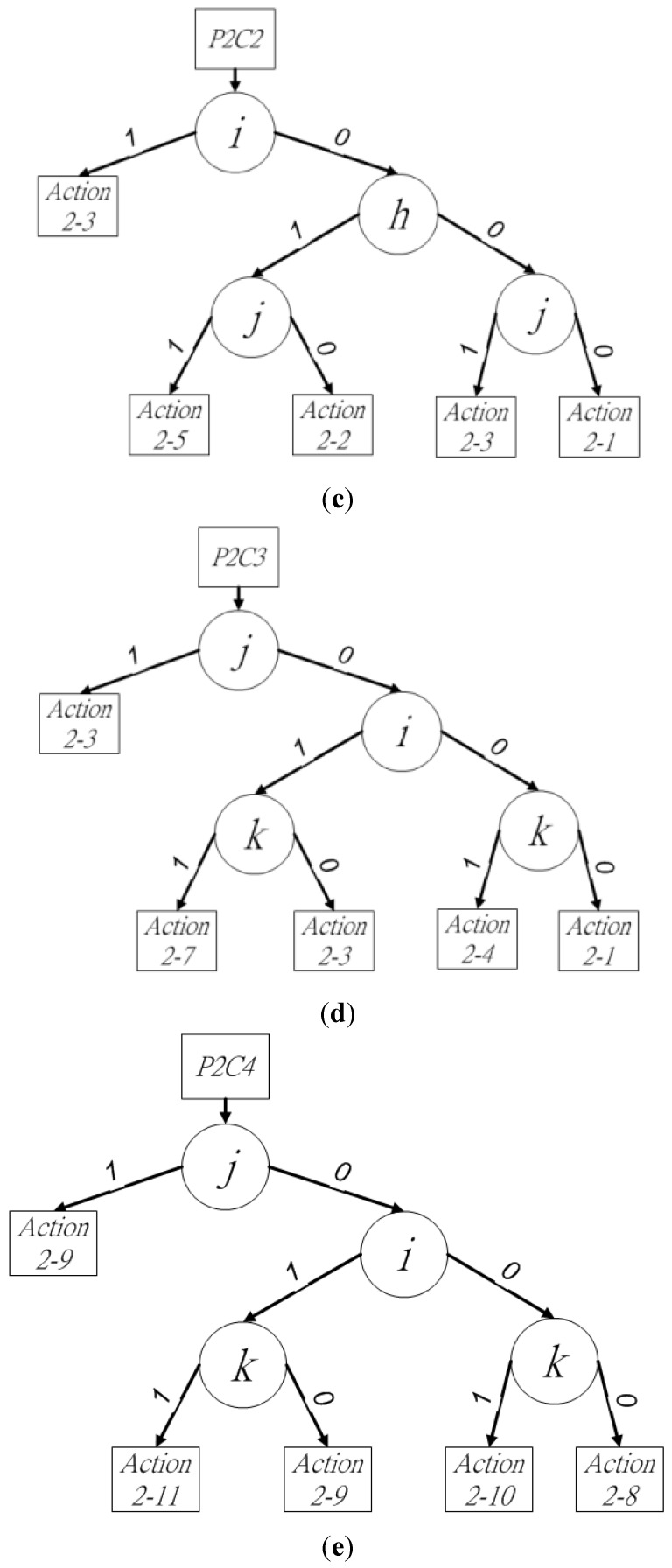
Binary decision tree of (**a**) procedure 2, (**b**) *P2C1*, (**c**) *P2C2*, (**d**) *P2C3*, and (**e**) *P2C4*.

The binary decision trees for procedure 2 are shown in Figure 6a. Pixels *o* and *p* determine four cases and affect the other three cases, because the existence of pixels *o* and *p* determines the connectivity between block *X* and adjacent blocks *P*, *Q*, and *R.* Thus, pixels *o* and *p* should be checked first to improve the labeling performance in block *X*. When (*op*)_2_ = (11)_2_ or (10)_2_, leaf nodes from *P2C1* or *P2C2* are chosen for further judgment. Pixel *s* is then checked when (*op*)_2_ = (01)_2_ or (00)_2_, because the connectivity between blocks *X* and *Q* must be ensured when pixel *o* is the background pixel. Leaf nodes from *P2C3*, *P2C4*, or *P2C5* are used for more judgments when (*ops*)_2_ = (011)_2_, (010)_2_, or (001)_2_. The final pixel to be checked in current block *X* by procedure 2 is *t*, which determines the selection of *P2C6* or *P2C7* by (*opst*)_2_ = (0001)_2_ or (0000)_2_. To summarize the seven cases and their leaf nodes, *P2C1*–*P2C4* have more judgments for block *X* and blocks *P*, *Q*, and *R* than the other cases in procedure 2. The binary decision sub-trees for these four cases are shown in Figure 6b–e. 

The seven cases of procedure 2 trigger 11 different actions as leaf nodes in the binary decision trees of procedure 2. The leaf nodes of the binary decision tree in procedure 2 include “assign the same provisional label as block *S* to block *X*,” “assign the same provisional label as block *S* to block *X*, and resolve the conflict among blocks *S* and *P*, *S* and *Q*, *S* and *R*, *S*, *P*, and *Q*, *S*, *P*, and *R*, or *S*, *Q*, and *R*,” “a new provisional label,” “assign the provisional label of block *P*, *Q*, or *R*,” and “assign the provisional label of block *P* or *Q*, and merge blocks *P* and *Q*, *P* and *R*, or *Q* and *R*.” The action numbers are summarized in Table 2.

**Table 2:** Actions of procedure 2.

Action Number	Neighbor Operations
2-1	Assign the same provision label as block S for the current block X
2-2	Assign the same provision label as block S for the current block X. Resolve the confliction with block P by assigning the provisional label of block S
2-3	Assign the same provision label as block S for the current block X. Resolve the confliction with block Q by assigning the provisional label of block S
2-4	Assign the same provision label as block S for the current block X. Resolve the confliction with block R by assigning the provisional label of block S
2-5	Assign the same provision label as block S for the current block X. Resolve the confliction with blocks P and Q by assigning the provisional label of block S
2-6	Assign the same provision label as block S for the current block X. Resolve the confliction with blocks P and R by assigning the provisional label of block S
2-7	Assign the same provision label as block S for the current block X. Resolve the confliction with blocks Q and R by assigning the provisional label of block S
2-8	Plus serial number and New a provisional label for block X as temporary label
2-9	Assign the same provision label as block Q for the current block X
2-10	Assign the same provision label as block R for the current block X
2-11	Assign the provisional label by block Q then merge blocks Q and R

In *P2C1*, pixels *o* and *p* are in state (11)_2_. The block-connected relationships between block *X* and blocks *S*, *P*, *Q*, and *R* should be checked, which requires pixels *h*, *i*, *j*, and *k* to be considered*.* The final action can be determined by following the binary decision sub-tree in Figure 6b. According to the locations of all foreground pixels, action numbers 2-1 to 2-7 can be used in the final decision. The first pixels to be checked are *i* and *j* to ensure the existence of block *Q*. The state of pixels *i* and *j* is (11)_2_, (10)_2_, (01)_2_, or (00)_2_, producing different actions for the current block *X*. When pixels *i* and *j* are in state (11)_2_, the block-connected relations among *X*, *P*, *Q*, and *R* are simplified to assign the provisional label for block S to block *X* and resolve the conflict between blocks *S* and *Q* as action 2-3. When pixels *i* and *j* are in state (10)_2_, the leaf node for the decision tree has two options, action 2-7 or 2-3, because blocks *S*, *Q*, and *R* may have different provisional labels. When pixel *k* is the foreground pixel, the neighbor operations assign the provisional label of block *S* to *X*, and resolve the conflict among blocks *S*, *Q*, and *R*. In contrast, action 2-3 assigns the provisional label of block *S* to *X*, and resolves the conflict between blocks *S* and *Q* when pixel *k* is 0*.* Either action 2-5 or 2-3 is triggered when pixels *i* and *j* are in state (01)_2_, because blocks *P* and *R* may not have the same provisional labels. Blocks *P* and *Q* must resolve their conflict via action 2-5 when pixel *h* is the foreground pixel. The next neighbor operation, action 2-5, assigns the provisional label of block *S* to *X*. Otherwise, action 2-3 is applied when pixel *h* is 0. Four different actions, actions 2-1, 2-2, 2-4, and 2-6, are triggered when pixels *i* and *j* are in state (00)_2_. The actions are determined by the four states of (*hk*)_2_. When (*hk*)_2_ is in state (11)_2_, blocks *Q* and *R* do not exist in the upper row of the current block *X*. Action 2-6 then assigns the provisional label of block *S* to block *X*, and resolves blocks *S*, *P*, and *R*. When the state of (*hk*)_2_ is (10)_2_, action 2-2 performs the neighbor operations of assigning the provisional label of block *S* to *X* and resolving the conflict between blocks *S* and *P*. When the state of (*hk*)_2_ is (01)_2_, block *R* exists and is connected to block *X*. Action 2-4 is used to assign the provisional label of block *S* to *X*, and resolve the conflict between block *S* and *R*. The final state of (*hk*)_2_ is (00)_2_. The neighbor operation assigns the provisional label of block *S* to block *X* as action 2-1 when blocks *P*, *Q*, and *R* do not exist. Pixels *s* and *t* are bypassed, because block *X* has already been assigned a provisional label by the leaf node of *P2C1*. Bypassing two checking pixels in procedure 2 eliminates three redundant cases. At the end of *P2C2*, the binary decision tree of the next judgment retains procedure 2, because pixel *p* is the foreground pixel representing the existence of block *Q*. 

Pixels *o* and *p* in state (10)_2_ represent *P2C2*. The block-connected relationships between block *X* and blocks *S*, *P*, and *Q* must be checked by pixels *h*, *i*, and *j.* Figure 6c shows the binary decision sub-tree. The middle pixel of pixels *h*, *i*, and *j* is checked first. Action 2-3 is performed, because pixels *h* and *j* have the same provisional label as *i* when pixel *i* is the foreground pixel. Otherwise, pixels *h* and *j* are checked when pixel *i* is 0. Pixels *h* and *j* have four different states that produce four different actions. Blocks *P* and *Q* may have different provisional labels when (*hj*)_2_ has the state (11)_2_. The leaf node performs action 2-5 as the neighbor operation. The second state of (*hj*)_2_ is (10)_2_, which indicates that block *P* is connected to block *X*. Action 2-2 is used to assign the provisional label of *S* to *X*, and resolve the conflict between *S* and *P*. The third state of (*hj*)_2_ is (01)_2_. Block *Q* is the only block in the upper row of block *X*. Action 2-3 is thus performed, assigning the provisional label of block *S* to *X* and resolving the conflict between blocks *S* and *Q*. The final state of (*hj*)_2_ is (00)_2_. Action 2-1 is used to assign the provisional label of block *S* to block *X*. The last neighbor operation of *P2C2* checks pixel *t* to determine which of procedures 1 and 2 should be used for the next judgment. Pixel *s* is bypassed, because the provisional label of block *X* has been acquired by following the binary decision tree. 

For *P2C3*, pixels *o*, *p*, and *s* are in state (011)_2_. The block-connected relationships between block *X* and blocks *S*, *P*, and *Q* are checked by pixels *h*, *i*, and *j.* Figure 6d shows the binary decision sub-tree. Pixel *j* must be checked first, because it is the middle pixel of *i*, *j*, and *k*. If pixel *j* is a foreground pixel, pixels *i* and *k* have the same provisional label. In this state, action 2-3 is applied to assign the provisional label of block *X*. Otherwise, pixels *i* and *k* are checked when pixel *j* is (0)_2_. The four different states of pixels *i* and *k* generate four different actions. The first state of (*ik*)_2_ is (11)_2._ Blocks *S*, *P*, and *Q* may have different provisional labels, which requires action 2-7. In the second state, (*ik*)_2_ is (10)_2_, whereby blocks *Q* and *X* are connected. Action 2-3 hence assigns the provisional label of *S* to *X* and resolves the conflict between *S* and *Q*. The third state of (*ik*)_2_ is (01)_2_. Block *R* is the only existing block in the upper row of block *X*. Action 2-4 thus assigns the provisional label of block *S* to *X* and resolves the conflict between blocks *S* and *R*. The final state of (*ik*)_2_ is (00)_2_, in which action 2-1 assigns the provisional label of block *S* to block *X*. The next judgment uses procedure 2, because pixel *s* is the foreground pixel. Pixel *t* is bypassed because the binary decision tree of the next judgment and the provisional label of block *X* have already been determined.

Pixels *o*, *p*, and *s* are in state (010)_2_ in *P2C4*. For block *X* and blocks *Q* and *R*, the block-connected relationships must be checked, which requires an examination of pixels *i*, *j*, and *k.* From Figure 6e, it is apparent that the current block *X* can determine the provisional label by following the binary decision sub-tree. In *P2C3*, the middle pixel of *i*, *j*, and *k* is first checked. However, blocks *S* and *X* have no connection with each other in *P2C4*. The assignment of the provisional label for block *X* by *Q* is applied as action 2-9 when pixel *j* is the foreground pixel. Pixels *i* and *k* are checked if pixel *j* is the background pixel. There are four different states for pixels *i* and *k*. The first state of (*ik*)_2_ is (11)_2_, where blocks *Q* and *R* may have different provisional labels. Action 2-11 is applied to the neighboring operations to assign the provisional label of block *Q* to *X* and resolve the conflict between blocks *Q* and *R*. The second state of (*hj*) _2_ is (10)_2_, whereby action 2-9 is applied to assign the provisional label of block *Q* to *X*. The third state of (*hj*)_2_ is (01)_2_. In this case, action 2-10 assigns the provisional label of block *R* to *X*. The binary decision tree for the next judgment chooses procedure 2, because pixel *p* is the foreground pixel. At the end of *P2C4*, pixel *t* is bypassed because it is connected to pixel *p*, and the provisional label has already been determined by the above process.

For *P2C5*, *P2C6*, and *P2C7*, the judgment focuses on the block-connected relationships of blocks *S* and *X*. In *P2C5*, pixels *s* and *t* have two states: (11)_2_ or (10)_2_. Action 2-1 is used to assign the provisional label of block *S* to block *X*. To select the correct procedure for the next judgment, pixel *t* must be checked to ensure the existence of block *S*. *P2C6* in procedure 2 has one foreground pixel at location *t*. Action 2-8 is thus performed to add and assign a new provisional label to block *X*. Pixel *t* has a value of 1, which ensures the next judgment retains the binary decision tree for procedure 2. *P2C7* involves no foreground pixels in block *X*. In this case, neighbor operations are avoided, and the binary decision tree of procedure 1 is chosen for the next judgment. The pseudo-code for procedure 2 of the first scan is given in Algorithm 2.

**Algorithm 2** Procedure 2 of the First-Scan Method in the Proposed Algorithm

**Begin**
1.    if(o!=0){
2.            if(p!=0)
3.            {P2C1;}
4.            else
5.            {P2C2;}
6.    }else if(p!=0)
7.            if(s!=0)
8.            {P2C3;}
9.            else
10.           {P2C4;}
11.   else if(s!=0)
12.           {P2C5;}
13.   else if(t!=0)
14.           {P2C6;}
15.   else
16.           {P2C7;}
**end**


### 3.3. Summary of Proposed Algorithm

At the end of the raster scan over an image, provisional labels are assigned to all foreground pixels by applying procedure 1 or procedure 2 in the first-scan method. Having collected all provisional labels, the second-scan method is used to sequentially update all provisional labels block by block to representative labels in the labeling table. In this paper, the data structures of the three equivalent arrays, the method of updating equivalent sets, and the second-scan method are the same as in the BBDT algorithm. The pseudo-code for combining procedures 1 and 2 in the first scan with the second scan is given in Algorithm 3.

**Algorithm 3** The Proposed Algorithm

**Begin**
1. Provisional label = 1;
2. for(y = 0; y<=H; y+=2) // begin the first-scan method
3.   for(x = 0; x<=W; x+=2) {
4.            Procedure1;
5.            while(keepinprocedure2==true&&x+2<W) {
6.                   x+=2;
7.                   Procedure2;
8.            }
9.       }
10. Update provisional labels to representative labels in the second-scan method;
**end**


## 4. Experimental Results and Analysis

Our comparison of experimental results from labeling algorithms focuses on previously proposed methods. Because the best techniques and structures available for labeling algorithms are those presented in [11] and [19], the proposed algorithm was compared with the Fast Connected-component Labeling (FCC) algorithm [11] and the Optimized Block-based Connected-component Labeling with Binary Decision Trees (BBDT) [19]. The source code of BBDT and the proposed algorithm are available online [25,28]. All three algorithms utilize the same data structures to process labeling jobs. The algorithms were implemented in C++ and compiled using Microsoft Visual Studio 2010 with identical commands. Our experiments were performed on an ASUS BM6660 using an Intel Core i7-2600 processor and 8 GB RAM with default frequency. Furthermore, the implemented algorithms produced identical numbers of labels and the same labeling results on all experimental datasets.

The BBDT algorithm is very efficient in labeling tasks. We implemented the BBDT algorithm ourselves, and compared the experimental results with those given in [19]. The implemented BBDT algorithm exhibited better speed and lower execution time than reported in [19]. In analyzing the source code of the BBDT algorithm, we found that the labeling speed was reduced by the OpenCV functions. The labeling task was slowed by the type transition from *byte* to *int* in the source code, and the use of multipliers for the image output array. Therefore, we modified the BBDT source code to remove the need to use OpenCV functions, and compared the resulting performance with that of the original BBDT algorithm. 

**Figure 7:**
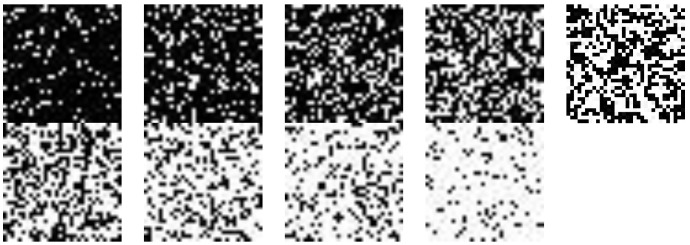
Examples of random noise images at a resolution of 32 × 32 that produced label densities from 0.1 to 0.9.

**Figure 8:**
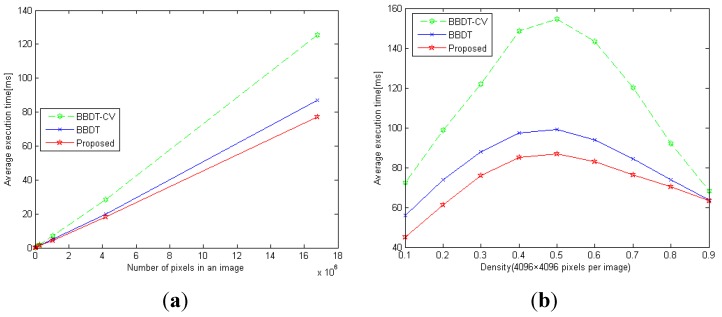
Execution time (ms) *versus* (**a**) the number of pixels in an image, and (**b**) the density of 4096 × 4096 pixel images.

We compared the proposed algorithm with the BBDT algorithms with and without OPENCV (referred to as BBDT and BBDT-CV, respectively) using a noisy dataset [25], as the examples shown in Figure 7. We utilized all the noisy images to test the linearity of the execution time with respect to the image size, as shown in Figure 8a. BBDT achieved lower execution times than BBDT-CV, by an average of 24.96%, and the proposed algorithm further reduced the execution time by 12.01% over that of BBDT. Moreover, the noise images with a size of 4096 × 4096 pixels were used to analyze the execution time with respect to the density of the image, as shown in Figure 8b. The execution time given for each density is the average execution time for ten images of that density. On average, BBDT reduced the execution time by 26.37% compared with BBDT-CV, and the proposed algorithm further reduced the execution time by 12.15% compared with BBDT. In particular, our algorithm reduced the execution time by an average of 42.82% over that of BBDT-CV for densities of 0.4–0.6. 

**Figure 9:**
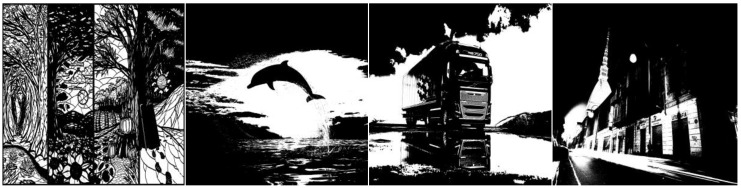
Image samples from the GF datasets.

Our evaluations focused on the labeling speed of the proposed algorithm, BBDT, and FCC using a real image dataset. The three algorithms were tested in two experiments. In the first experiment, images were taken from the “GdeFon” (GF) datasets [29]. Sixty images were selected and resized to 6000 × 6000, 7000 × 7000, 8000 × 8000, 9000 × 9000, 10,000 × 10,000, 11,000 × 11,000, and 12,000 × 12,000 pixels per image. The downloaded images were taken from diverse categories such as “abstraction,” “animal,” “auto,” “city,” “holiday,” “landscapes,” “space,” and “stuff” to provide a large number of provisional labels, as shown in Figure 9. All images were transformed into grayscale by Otsu’s method [30] prior to the experiments.

**Figure 10:**
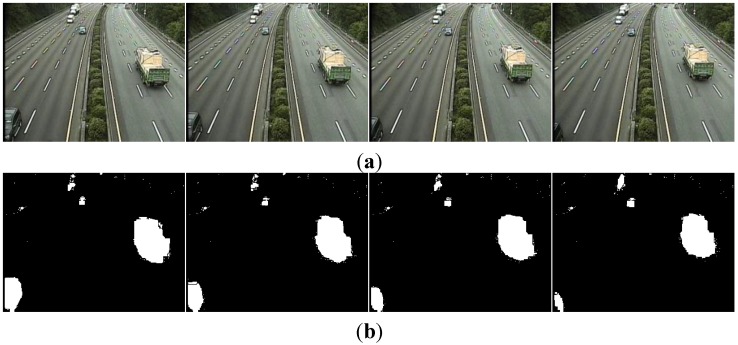
Image samples from the FH dataset. (**a**) Original images, and (**b**) foreground images.

The second experiment used 382 sequential images of real driving scenarios containing various sizes and types of vehicles from a dataset that we call “Formosa Highway” (FH) in this paper. The FH image dataset [31] was captured using a camera on the Formosa Highway in Taiwan. The FH image dataset shows a surveillance perspective of parts of the highway. The sequential images of the FH dataset have a size of 320 × 240 pixels each. Prior to applying the labeling algorithms, the images were pre-processed according to the background subtraction method in [32] to generate foreground pictures. Eight sample images from the FH dataset are shown in Figure 10.

**Figure 11:**
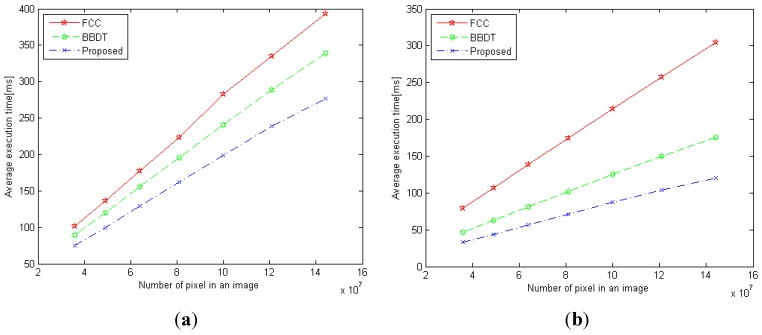
Average execution time for each of the three labeling algorithms *versus* the scaling image size: (**a**) the entire algorithm, and (**b**) the first-scan method.

The GF dataset was used to test the average execution time of each algorithm against the number of pixels in each image. The average time required by each of the three labeling algorithms, as well as that of their first-scan methods, is shown in Figure 11. The results show that the average execution time for all three algorithms exhibited linear characteristics. The proposed algorithm outperformed the other two labeling algorithms for all image sizes. For images of size 12,000 × 12,000 pixels, the execution times of our proposed algorithm, BBDT, and FCC were 276.72 ms, 338.83 ms, and 392.93 ms, respectively, as shown in Figure 11a. Thus, our algorithm was 18.33% and 29.57% faster than the BBDT and FCC algorithms, respectively. With regard to the first-scan method, the proposed algorithm, BBDT, and FCC recorded average execution times of 120.66 ms, 175.92 ms, and 304.44 ms, respectively, to complete the labeling task, as shown in Figure 11b. The proposed algorithm thus required 31.41% and 60.36% less time than the BBDT and FCC algorithms, respectively. According to the experimental results for the GF dataset, the proposed algorithm is the most efficient of the three labeling algorithms tested.

**Figure 12:**
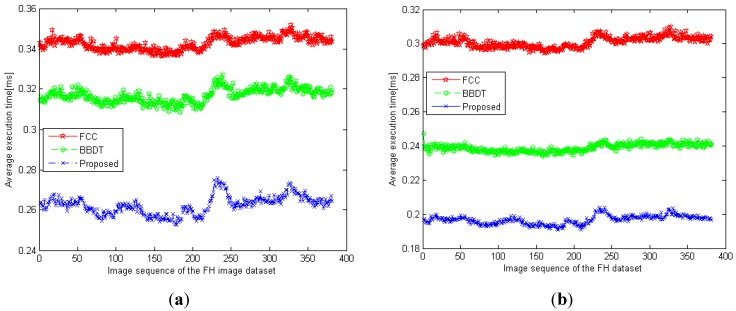
Average execution times of the three labeling algorithms *versus* the image sequence of the FH image dataset: (**a**) the entire algorithm, and (**b**) the first-scan method.

The execution times of the three algorithms were then tested using the surveillance images of vehicles on Formosa Highway. The average execution time of each of the three labeling algorithms on the FH dataset is shown in Figure 12**,** where the value for each point is the average execution time per image. The proposed algorithm attained lower execution time than the other two labeling algorithms for the FH dataset. As shown in Figure 12a, the average execution times of the proposed algorithm, BBDT, and FCC were 0.262 ms, 0.317 ms, and 0.344 ms, respectively, for the 320 × 240 image resolution. The proposed algorithm thus reduced the execution time by 17.35% and 23.84% over that of the BBDT and FCC algorithms, respectively. Furthermore, the execution times of the first-scan method for the proposed algorithm, BBDT, and FCC were 0.197 ms, 0.239 ms, and 0.301 ms, respectively, for all 382 images, as shown in Figure 12b. The proposed algorithm was therefore 17.57% and 34.55% faster than the BBDT and FCC algorithms, respectively, in the first-scan process. Based on the experimental results from the FH dataset, the proposed algorithm is the fastest of the three labeling algorithms.

To analyze the memory usage, we compared the number of leaf nodes generated and the depth of the decision trees (e.g., the access frequency of the merge decision for each pixel). The BBDT algorithm contained 211 leaf nodes with 14 levels for the binary decision tree, Sutheebanjard *et al.* [21] improved decision tree based on the BBDT algorithm used 86 leaf nodes with 12 depth levels, and our proposed algorithm generated two binary decision trees in procedures 1 and 2 using the simplified block-based scan mask. In procedure 1, the binary decision tree contained three sub-trees and three leaf nodes with five depth levels. *P1C1* contained nine leaf nodes with five depth levels connecting the main binary decision tree. There were five leaf nodes with four depth levels for the binary decision trees of *P1C2* and *P1C3*, which were connected to the main binary decision tree. The binary decision tree for procedure 1 contained 22 leaf nodes with seven depth levels. The binary decision tree for procedure 2 contained four sub-trees and three leaf nodes with five depth levels. *P2C2* contained nine leaf nodes with five depth levels connecting the binary decision tree. There were five leaf nodes with four depth levels for the binary decision trees of *P2C2*, *P2C3*, and *P2C4* connected to the binary decision tree. The binary decision tree for procedure 2 contained 27 leaf nodes with seven depth levels. To summarize, our algorithm generated 49 leaf nodes with seven depth levels for the two binary decision trees. Hence, the proposed algorithm used 43.02% fewer leaf nodes and 41.67% fewer depth levels for the decision trees. Furthermore, the proposed algorithm generated 76.77% fewer leaf nodes and 50% fewer depth levels than the BBDT algorithm. Thus, the proposed algorithm is more efficient in terms of memory usage than the algorithm proposed by Sutheebanjard *et al.* [21] and BBDT [19] in terms of leaf nodes and depth levels in the decision trees.

## 5. Conclusions

In this paper, we have proposed an efficient scanning algorithm for block-based connected-component labeling. The proposed method is expected to assist the labeling process in the field of computer vision. Using eight pixels selected from the original 20 pixels in the block-based scan mask, we developed a simplified block-based scan mask using efficient strategies involving two procedures in different scenarios. The two procedures are efficiently combined into the first-scan method, and are processed using binary decision trees through the simplified block-based scan mask. Compared with the FCC and BBDT algorithms, our proposed method enables improved labeling performance and execution time. Experimental results showed that the proposed algorithm is superior to the FCC algorithm and the BBDT algorithm.
